# Polymeric Particle BAM15 Targeting Macrophages Attenuates the Severity of LPS-Induced Sepsis: A Proof of Concept for Specific Immune Cell-Targeted Therapy

**DOI:** 10.3390/pharmaceutics15122695

**Published:** 2023-11-28

**Authors:** Kanyarat Udompornpitak, Thansita Bhunyakarnjanarat, Wilasinee Saisorn, Chonnavee Manipuntee, Kittawat Plengplang, Samarch Sittichaitaweekul, Panisa Jenphatanapong, Suwasin Udomkarnjananun, Warerat Kaewduangduen, Kasirapat Ariya-anandech, Amanee Samaeng, Numpon Insin, Patcharee Ritprajak, Asada Leelahavanichkul

**Affiliations:** 1 Department of Microbiology, Faculty of Medicine, Chulalongkorn University, Bangkok 10330, Thailand; jubjiibb@hotmail.com (K.U.);; 2 Center of Excellence in Translational Research on Immunology and Immune-Mediated Diseases (CETRII), Department of Microbiology, Faculty of Medicine, Bangkok 10330, Thailand; 3 Interdisciplinary Program of Biomedical Sciences, Graduate School, Chulalongkorn University, Bangkok 10330, Thailand; 4 Department of Chemistry, Faculty of Science, Chulalongkorn University, Bangkok 10330, Thailand; 5 Research Unit in Integrative Immuno-Microbial Biochemistry and Bioresponsive Nanomaterials, Faculty of Dentistry, Chulalongkorn University, Bangkok 10330, Thailand; porpluemw@gmail.com (W.K.);; 6 Division of Nephrology, Department of Medicine, Faculty of Medicine, Chulalongkorn University and King Chulalongkorn Memorial Hospital, Bangkok 10330, Thailand; suwasin.u@gmail.com; 7 Department of Microbiology, Faculty of Dentistry, Chulalongkorn University, Bangkok 10330, Thailand

**Keywords:** BAM15, PLGA particle, targeting, macrophage, sepsis, LPS

## Abstract

Macrophage polarization requires different energy sources and metabolic processes. Therefore, cell energy interference to alter macrophage functions has been proposed as a treatment for severe inflammatory diseases, including sepsis. In this study, targeting cell energy using BAM15 (a mitochondrial uncoupling agent) in human THP-1 and mouse RAW264.7 macrophages prominently interfered with M1 but not M2 polarization. Free BAM15 (BAM15) and BAM15-loaded PLGA particles (BAM15 particles) reduced the inflammatory response of M1 macrophages and enhanced the expression of M2 signature genes with the restoration of mitochondrial activity (extracellular flux analysis) in RAW264.7 cells. Furthermore, BAM15 particles but not BAM15 showed specific effects on the inflammatory response of macrophages but not neutrophils, and the particles were actively captured by splenic and liver macrophages in vivo. Administration of BAM15 and BAM15 particles attenuated the severity of sepsis in LPS-induced sepsis mice. Interestingly, BAM15 particles but not BAM15 alleviated LPS-induced liver injury by reducing hepatic inflammation. Our findings substantiate the superior efficacy of macrophage-targeted therapy using a BAM15 particle-delivery system and provide further support for clinical development as a potential therapy for severe inflammatory diseases.

## 1. Introduction

Macrophages are innate immune cells, and the plasticity of their phenotypes and functions are important in several diseases [1,2,3]. Over the past decade, regulating macrophage polarization has become a potential strategy in the treatment of immune-related diseases, such as sepsis, autoimmunity, and cancer. Blockage of pro-inflammatory M1 macrophage polarization attenuates overwhelming inflammation in severe infection and autoimmune diseases [2,4], while reduction in anti-inflammatory M2 macrophage polarization enhances inflammatory responses that are necessary for controlling malignant cells [5,6]. The polarization between M1 and M2 macrophages requires different energy sources and metabolic pathways. As such, a glycolytic burst and the pentose phosphate pathway (PPP) are necessary for rapid energy production during M1 macrophage polarization, while oxidative metabolism is mainly required for M2 macrophage polarization [3,7]. In addition, adjustments in mitochondrial metabolism, including changes in oxidative metabolism, mitochondrial reactive oxygen species (mtROS), mitochondrial ultrastructure, and membrane potential, contribute to different states of macrophage activation [8,9]. Hence, the interference of macrophage energy metabolism (glycolysis and mitochondrial activities) alters macrophage phenotypes and functions, which consequently affect host immune responses [10]. 

Mitochondrial uncoupling is a process that affects cellular bioenergy and the production of reactive oxygen species (ROS). Recent evidence has shown the importance of mitochondrial uncoupling in the regulation of macrophage activities and immune responses [11]. Accordingly, BAM15 (N^5^, N^6^-bis(2-fluorophenyl) (2,1,3) oxadiazolo (4,5-b) pyrazine-5,6-diamine) is a synthetic mitochondrial uncoupler that shows anti-obesity and anti-cancer effects [12,13]. Our previous studies demonstrated that BAM15 alleviates macrophage inflammatory responses in an LPS-induced sepsis mouse model. Despite less toxicity than other mitochondrial uncoupling agents, the toxicity of BAM15 has been reported, especially in high doses, perhaps due to profound mitochondrial blockage [14]. Thus, attempts to reduce the side effects of this agent are of great importance. As such, a particle-based drug delivery system specifically targeting macrophages is likely to reduce off-target BAM15 toxicity [15,16]. 

Poly (D, L-lactide-coglycolide) (PLGA) particles are one of the interesting polymeric biodegradable particles that have been used as a carrier for the delivery of drugs and biomolecules. Due to its biocompatible and biodegradable properties, PLGA particles have been approved by the United States (US) Food and Drug Administration (FDA). Additionally, PLGA particles can destabilize endosomes, induce endosomal escape, and deliver cargo to specific target cells [17,18]. We previously developed PLGA-based particles for drug delivery to dendritic cells (DCs) [19,20], while the delivery to macrophages in a similar aspect is also interesting. Therefore, BAM15-loaded PLGA may be an interesting anti-inflammatory drug for the treatment of several pro-inflammatory conditions. 

Sepsis is a potentially fatal inflammatory reaction to a systemic infection that is a significant global health problem with a complex interplay between several mechanisms, including immunological homeostasis, elevated reactive oxygen species levels, and insufficient tissue perfusion [21,22]. The mortality rate for sepsis remains high, despite improved supportive treatment, which is partly due to the hyper-responses to microbial molecules of several immune cells, especially macrophages [23]. Indeed, the immunological blockage on macrophages using LysM-Cre mice [24] and nanomaterials targeting macrophages successfully attenuates hyper-inflammation in sepsis [25,26]. Here, several tests were performed to assess (i) the impacts of BAM15 on lipopolysaccharide (LPS)-induced M1 polarization or M2 polarization (using IL-4 and cancer cell supernatant) and (ii) the influence of BAM15-PLGA particles (BAM15 particles) on macrophages and in mice. These analyses were conducted to explore the possible use of BAM15 particles in targeted drug delivery for the treatment of immune-mediated inflammatory diseases. 

## 2. Materials and Methods

### 2.1. Animals and Animal Model

The animal care and use protocol was approved by the Institutional Animal Care and Use Committee of the Faculty of Medicine, Chulalongkorn University, Bangkok, Thailand (SST10/2561), in accordance with the US National Institutes of Health standard. C57BL/6 mice (8 weeks old) were purchased from Nomura Siam International (Bangkok, Thailand). Moreover, N5, N6-bis (2-fluorophenyl) (2,1,3) oxadiazolo (4,5-b) pyrazine-5,6-diamine (BAM15) (Sigma-Aldrich, St. Louis, MO, USA) at 2 mg/kg [12], BAM15 particles (with a dose of BAM15 of 2 mg/kg), blank particles (with the same amount of particles as the groups administered BAM15 particles), or phosphate buffer solution (PBS) (untreated group) were administered intraperitoneally at 0.5 h before intraperitoneal injection of lipopolysaccharide (LPS) from *Escherichia coli* 026:B6 (Sigma-Aldrich) (4 mg/kg in PBS) [27]. Notably, BAM15 was prepared in 10% dimethyl sulfoxide (DMSO) before mixing with PBS. Blood samples were collected by tail-vein nicking at 0.5 and 1 h after LPS injection. At 2 h after LPS injection, blood and organs (kidneys and livers) were collected under isoflurane anesthesia. Organs were weighed and thoroughly sonicated (High-Intensity Ultrasonic Processor, Newtown, CT, USA) in a 500 mL PBS-containing protease inhibitor cocktail (I3786; Sigma-Aldrich). The tissue lysates were centrifuged, and the supernatants were collected. The cytokines (TNF-α, IL-6, and IL-10) from serum and tissue lysates were quantified by the enzyme-linked immunosorbent assay (ELISA). Serum creatinine was measured with the QuantiChrom Creatinine Assay (DICT-500) (BioAssay). Aspartate transaminase (AST) and alanine transaminase (ALT) levels were measured with EnzyChrom AST (EASTR-100) and EnzyChrom ALT (EALT-100) (BioAssay, Hayward, CA, USA), respectively.

### 2.2. Preparation of Poly Lactic-Co-Glycolic Acid Particles (PLGA)

PLGA particles were synthesized using a single emulsion/solvent evaporation [19,20]. Briefly, 50:50 lactide:glycolide PLGA (Sigma-Aldrich) (100 mg) in dichloromethane (10 mL) (RCI Labscan, Bangkok, Thailand) was homogenized for 2 min before the organic solvent was evaporated at 25 °C with magnetic stirring (2 h). A particle size of 500 nm was acquired using stepwise centrifugation at various centrifugal forces. Briefly, the obtained particles were centrifuged at 500 relative centrifugal force (rcf) to remove particles that were too large before centrifugation of the supernatant (2000 rcf) to obtain 500 nm particles (blank particles). For BAM15-incorporated PLGA (BAM15 particles), the mixture of BAM15 (1 mg in 1 mL methanol) with blank particles (5 mg in 4 mL water) was centrifuged at 2500 rcf. Then, free BAM15 and BAM15 particles were measured in the supernatant and sediment, respectively, and a standard curve of serially diluted BAM15 was obtained using a UV-VIS spectrophotometer [28] at an absorbance of 330 nm (peak of BAM-15, Figure S1) and 600 nm (baseline). The amounts of incorporated BAM15 were calculated by subtracting the free BAM15 from the initial amounts of BAM15. Here, 94 µg of BAM15 was incorporated into 1 mg of PLGA. 

For particle characterization, PLGA and BAM15 particles were processed with gold sputter coating. The morphology and size of the particles were determined using a scanning electron microscope (SEM) (JSM-IT100, JEOL, Tokyo, Japan). The hydrodynamic diameter, polydispersity indices (PDIs) and zeta potential parameters were measured using a Zetasizer (ZSP; Zetasizer, Malvern Instruments Ltd., Malvern, UK). All measurements were made in Milli-Q water.

### 2.3. In Vivo Uptake of Fluorescein Isothiocyanate (FITC)-Tagged Particles (PLGA-FITC)

To trace the particles in mice, FITC-tagged particles (PLGA-FITC) were prepared. As such, FITC (Sigma-Aldrich) (500 µg in 1 mL ethanol) and PLGA (5 mg) dispersed in 400 µL water were stirred for 1 h at room temperature before washing the free fluorescence with water; the mixture was stored in the dark at 4 °C until use. On the other hand, 1 mg of PLGA-FITC or unlabeled PLGA particles was intraperitoneally injected into mice. The spleens and livers were collected at 24 h post-injection under isoflurane anesthesia. Subsequently, the organs (liver and spleen) were minced and digested with collagenase IV (300 units/mL) (Sigma-Aldrich) and DNaseI (10 units/mL) (Invitrogen, ThermoFisher Scientific, Waltham, MA, USA) at 37 °C with shaking at 150 revolutions per minute (rpm) for 30 min. The homogenized cells were re-suspended in Roswell Park Memorial Institute (RPMI) media and passed through cell strainers. Red blood cells were lysed with red blood cell lysis buffer (ammonium chloride; NH4Cl) (Merck KGaA, Darmstadt, Germany), and the samples were washed with PBS. The splenic and liver cell suspension were preincubated with the Fc region blocking agent (BioLegend, San Diego, CA, USA) for 10 min. Subsequently, cells were stained with fluorescence-tagged monoclonal antibodies specific for mouse F4/80 (clone BM8; BioLegend) and mouse CD11c (clone N418; BioLegend) for 20 min. The cells were washed and then fixed with 4% paraformaldehyde. All stained cells were evaluated by flow cytometry (BD LSRII cytometer, BD Biosciences, Franklin Lakes, NJ, USA). The flow cytometry data were analyzed using FlowJo 10.8 software (BD Biosciences). Additionally, fluorescence imaging analysis was performed following previous publications [29]. Briefly, organs were prepared in Tissue-Tek O.C.T Compound (Sakura Finetek, CA, USA) and stained with DAPI (4′,6-diamidino-2-phenylindole) (Abcam, Cambridge, MA, USA). Then, the images were visualized, and the fluorescence intensity was analyzed using ZEISS LSM 800.

### 2.4. Human Cell Line Experiments

Human leukemia monocytic cells of the THP-1 cell line (TIB-202 ^TM^; ATCC, Manassas, VA, USA) were cultured in RPMI 1640 (Invitrogen, ThermoFisher Scientific, Waltham, MA, USA) supplemented with 10% heat-inactivated fetal bovine serum (Invitrogen), 100 U/mL penicillin, 100 µg/mL streptomycin (Invitrogen), and 50 µM 2-mercaptoethanol. After that, THP-1 cells were incubated with 100 ng/mL of phorbol 12-myristate 13-acetate (PMA) (Sigma-Aldrich) for macrophage differentiation [30]. Then, the differentiated THP-1 cells were stimulated with RPMI (untreated), LPS from *E. coli* 026:B6 (100 ng/mL) (Sigma-Aldrich), interleukin 4 (IL-4) (20 ng/mL), and 20% tumor-conditioned media prepared by centrifuging 5 × 10^6^ CaCO2 and HepG2 cells (cancer cell lines) incubated in the presence or absence of BAM15 (50 ng/mL) [31]. At 24 h after stimulation, supernatants were collected for cytokine quantification using ELISA (Invitrogen), and the cells were harvested for quantitative real-time polymerase chain reaction (PCR).

### 2.5. Mouse Cell Line Experiments

Murine macrophages (RAW264.7) (TIB-71^TM^; ATCC) were cultured in Dulbecco’s Modified Eagle Medium (DMEM; Invitrogen) supplemented with 10% heat-inactivated fetal bovine serum (Invitrogen), 100 U/mL penicillin, and 100 µg/mL streptomycin (Invitrogen). On the other hand, neutrophils were isolated from mouse blood using an established gradient separation protocol [32]. Briefly, blood (1 mL) was added to 3 mL of Ficoll-Paque Polymorprep (Axis-Shield, Dundee, UK) and centrifuged for layer separation before neutrophil collection with lysis of red blood cells (NH4Cl). The isolated neutrophils were cultured in modified RPMI 1640 (Invitrogen). The cells were stimulated with culture media (untreated), BAM15 (free BAM15), BAM particles (with an equal amount of the loaded BAM15 to free BAM15), and blank particles (with an equal amount of the particles compared to the BAM15 particles) in the presence or absence of LPS (100 ng/mL). At 24 h after stimulation, supernatants were collected for cytokine quantification using ELISA (BioLegend), and the cells were harvested for quantitative real-time PCR. Additionally, the cell viability test was performed using the MTS assay (CellTiter 96^®^ AQueous One Solution Cell Proliferation Assay; Promega, Madison, WI, USA) at 24 h post-stimulation. Furthermore, neutrophil chemotaxis, as indicated by the number of cells that migrated from the upper chamber to the lower part of a 3 µm Transwell system (Millipore, Burlington, MA, USA), were determined using an automated cell counter (CountessTM II) (Thermo Fisher Scientific).

### 2.6. Quantitative Real-Time PCR

RNA was extracted from cell pellets using a FarvoPrep RNA mini kit (Farvogen, Vienna, Australia). The amounts of RNA were quantified using a NanoDrop OneC Microvolume UV-Vis Spectrophotometer, and cDNA was synthesized using a cDNA reverse transcription kit (Applied Biosystems, Warrington, UK). Quantitative PCR was performed using SYBR^®^ Green PCR Master Mix and an Applied Biosystems QuantStudio 6 Flex Real-Time PCR System (Applied Biosystems). The results were demonstrated in terms of the relative quantification of the comparative threshold method (delta-delta Ct; 2^−ΔΔCt^) as normalized by *ATCB* (β-actin) (an endogenous housekeeping gene). The primers for the target genes are listed in Table 1.

### 2.7. Analysis of Cell Energy Metabolism

Extracellular flux analysis (cell energy status) of 24 h-activated macrophages with the above conditions were measured with Seahorse XFp Analyzers (Agilent, Santa Clara, CA, USA). The oxygen consumption rate (OCR) and the extracellular acidification rate (ECAR) represent mitochondrial function (respiration) and glycolysis activity, respectively. For cell energy intervention, several agents, including glucose, oligomycin, 2-deoxy-D-glucose (2-DG), and rotenone/antimycin A, were injected sequentially according to the manufacturer’s protocol [27]. The calculation of maximal respiration with Seahorse Wave 2.6 software was based on the OCR between FCCP and rotenone/antimycin A subtracted by the OCR after rotenone/antimycin A. The area under the curve of the ECAR was based on the trapezoidal rule. 

### 2.8. Statistical Analysis

The mean ± standard error of the mean (SEM) was determined using one-way analysis of variance (ANOVA) followed by Tukey’s honest significant difference (HSD) analysis for multiple group comparisons. The analysis of the time-point data was determined with repeated measures ANOVA. All statistical analyses were performed with Graph Pad Prism version 10.0 software (La Jolla, CA, USA), and a *p*-value of <0.05 was considered statistically significant.

## 3. Results

### 3.1. BAM15 Interfered with the Polarization of M1 but Not M2 Macrophages in a Human Macrophage Cell Line

**Figure 1:**
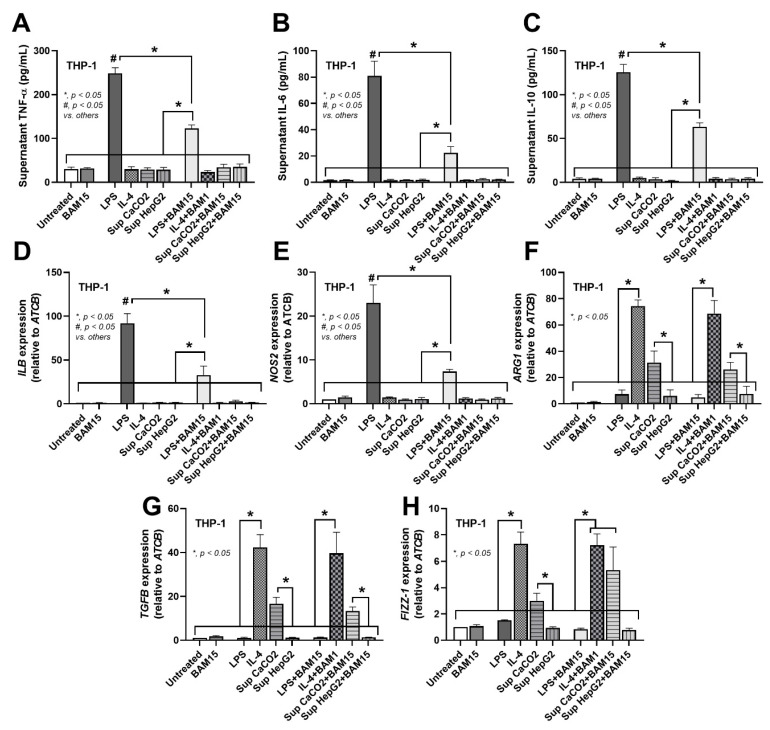
Characteristics of macrophages (differentiated THP-1 cells) were examined at 24 h after stimulation with culture media (untreated), LPS, IL-4, and supernatant of HepG2 and CaCO2 cancer cells in the presence or absence of BAM15. (**A**–**C**) The supernatant cytokines TNF-α, IL-6, and IL-10 were quantified by ELISA. (**D**–**H**) The expression of M1 genes (*IL1B* and *NOS2*) and M2 genes (*ARG1*, *TGFB*, and *FIZZ1*) was determined by quantitative real-time PCR. The results were derived from three independent experiments. * *p* < 0.05 between the indicated groups.

Human monocyte cells of the THP-1 cell line were differentiated into macrophages with phorbol 12-myristate 13-acetate (PMA) (THP-1 macrophage) [30], and then the cells were polarized to M1 and M2 macrophages in the presence or absence of BAM15. As such, LPS was used to induce M1 polarization as indicated by supernatant cytokines (TNF-α, IL-6, and IL-10) (Figure 1A–C) and M1 polarization genes (*IL1B* and *NOS2*) (Figure 1D,E) with low expression of M2 polarization genes (*ARG1*, *TGFB*, and *FIZZ1*) (Figure 1F–H). With BAM15, all M1 cytokines and genes of LPS-stimulated THP-1 macrophages were suppressed (Figure 1A–E). In parallel, IL-4 was used to induce a more profound M2 polarization than the use of CaCO2 supernatant as indicated by the higher expression of M2 genes (*ARG1*, *TGF-β*, and *FIZZ1*), while HepG2 supernatant could not induce M2 polarization (Figure 1F–H). There was no difference in the expression of M2 genes in THP-1 macrophages treated with IL-4 or supernatants of CaCO2 and HepG2 cells with BAM15 versus without BAM15 (Figure 1F–H). The data suggest that BAM15 potentially inhibited the polarization of pro-inflammatory M1 macrophages, but it did not affect anti-inflammatory M2 macrophage polarization.

### 3.2. BAM15 Altered Mitochondrial Energy Metabolism in M1 Macrophages

**Figure 2:**
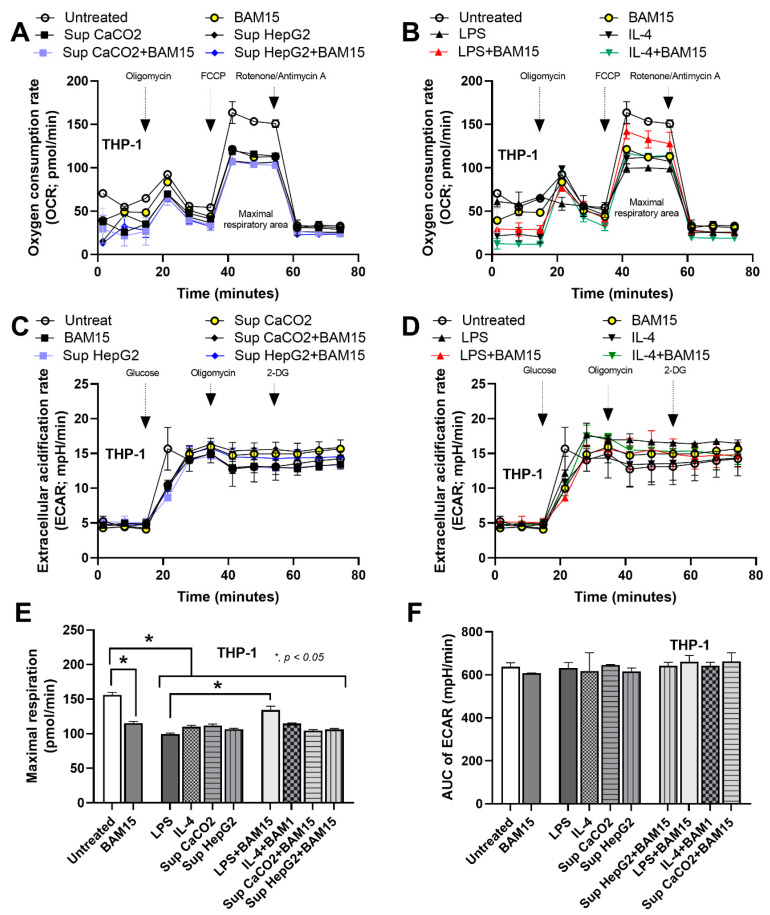
The cell energy metabolism of macrophages (differentiated THP-1 cells) was measured at 24 h after stimulation with culture medium (untreated), lipopolysaccharide (LPS), IL-4, HepG2, and CaCO2 cancer cells in the presence or absence of BAM15. (**A**,**B**) Oxygen consumption rate (OCR), (**C**,**D**) extracellular acidification rate (ECAR), (**E**) maximal respiratory rate, and (**F**) area under the curve (AUC) of ECAR were measured to indicate the energy status and glycolytic activity of the cells. The results were derived from three independent experiments. * *p* < 0.05 between the indicated groups.

Mitochondrial energy metabolism was measured in THP-1 macrophages with M1 and M2 induction in the presence or absence of BAM15 (Figure 2). A decrease in the oxygen consumption rate (OCR) and maximal respiratory rate during M1 and M2 polarization (Figure 2A,B,E) indicated a reduction in cell energy production. However, glycolysis measured by the extracellular acidification rate (ECAR) and area under the curve of ECAR (AUC of ECAR) did not change during M1 and M2 polarization (Figure 2C,D,F). Interestingly, BAM15 treatment enhanced the OCR in LPS-induced M1 polarization, but it did not affect the OCR in M2 macrophages induced by IL-4 and cancer cell supernatants (Figure 2A,B). Moreover, BAM15 treatment did not alter glycolysis in both M1 and M2 macrophages (Figure 2C,D,F). Consistent with the OCR data, BAM15 treatment enhanced the maximal respiration in M1 macrophages but not M2 macrophages. Therefore, BAM15 may be useful in reducing hyperinflammatory responses of M1-polarized macrophages by altering the energy status of M1.

### 3.3. BAM15 and BAM15-PLGA Particles (BAM15 Particles) Attenuated Inflammatory Responses in LPS-Activated Macrophages but Not Neutrophils

**Figure 3:**
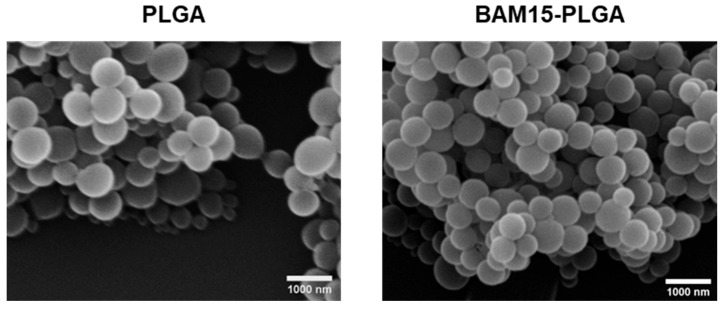
Sizes and morphologies of particles. Scanning electron microscopy (SEM) image of PLGA and BAM15-loaded PLGA (BAM15-PLGA) particles. Average size of PLGA is 515 ± 46 nm, and average size of BAM15-PLGA is 529 ± 52 nm.

Next, we delivered BAM15 in vitro using the PLGA particle platform. It has been reported that macrophages favor a particle size ranging from 200 to 1000 nm [33,34]; therefore, we selected a particle size of 500 nm and attached BAM15 to the surface of the particles. The morphology and size of PLGA (blank particles) and BAM15-PLGA particles (BAM15 particles) were confirmed by SEM analysis. Both particles exhibited spherical shapes with sizes of 515 ± 46 nm and 529 ± 52 nm, respectively (Figure 3). The hydrodynamic size of the PLGA particles (527 ± 6) and the BAM15 particles (559 ± 17) corresponded to the SEM data (Table 2 and Figure 3). In addition, the sizes of both particles were uniform, as indicated by the polydispersity index (PDI) (Table 2). The zeta potential values of the PLGA and BAM15-PLGA particles were −36.1 ± 0.3 mV and −30.1 ± 0.5, respectively, indicating that BAM15 was attached to the PLGA particles (Table 2).

**Figure 4:**
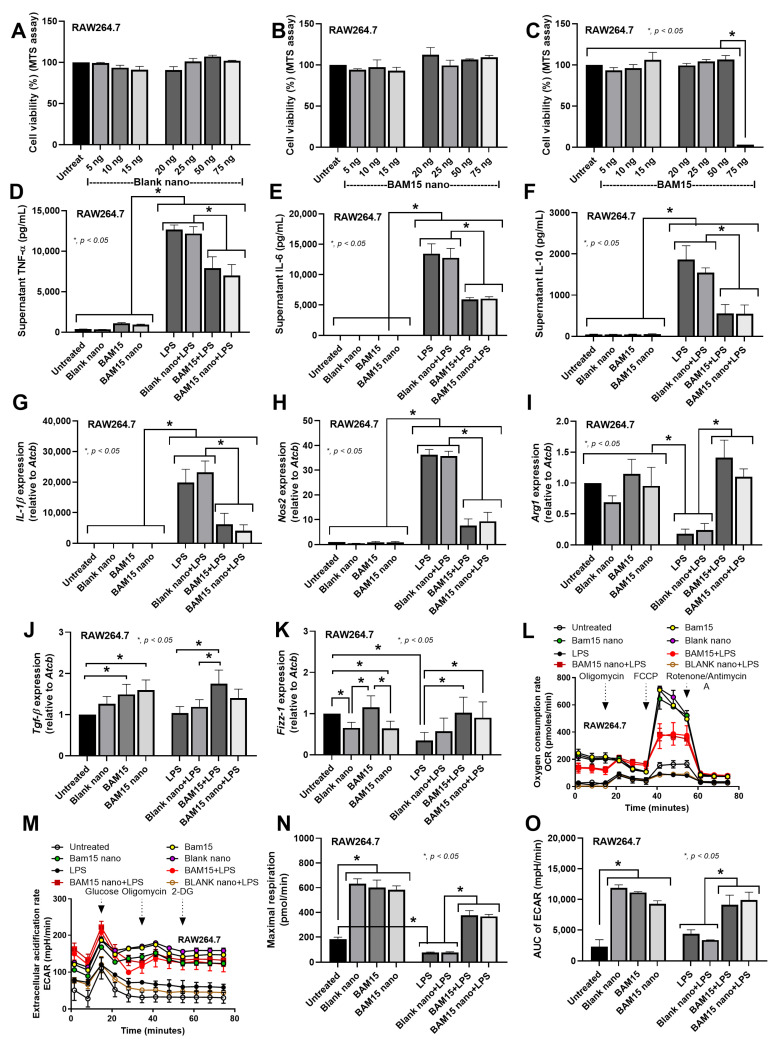
(**A**–**C**) Cytotoxicity tests in RAW264.7 macrophages. The RAW264.7 macrophages were incubated with 5–75 ng of free BAM15 (BAM15), BAM15-PLGA particles (BAM15 particles) (BAM15 prts) containing 5–75 ng of BAM15, and blank particles (with the same amounts as BAM15 particles) for 24 h. The untreated cells were used as the negative control. The characteristics of RAW264.7 macrophages were demonstrated 24 h after activation with BAM15 (50 ng), BAM15 particles (containing 50 ng of BAM15), and blank particles (with the same amounts to BAM15 particles) in the presence or absence of LPS. (**D**–**F**) The supernatant cytokines TNF-α, IL-6, and IL-10 were quantified with ELISA. (**G**,**H**) The expression of M1 genes (*Il1B* and *Nos2*) and M2 genes (*Arg1*, *Tgfb*, and *Fizz1*) was determined by quantitative real-time PCR. (**L**–**O**) Cell energy metabolism of RAW264.7 macrophages was measured at 24 h after activation with culture medium (untreated), lipopolysaccharide (LPS), IL-4, HepG2, and CaCO2 cancer cells in the presence or absence of BAM15. The oxygen consumption rate (OCR), the extracellular acidification rate (ECAR), the maximal respiratory rate, and the area under the curve (AUC) of the ECAR were measured to indicate the energy status and glycolytic activity of the cells. The results were derived from three independent experiments. * *p* < 0.05 between the indicated groups.

The cytotoxicity test with the MTS assay demonstrated that blank particles and BAM15 particles with all selected BAM15 doses (5–75 ng/well) did not affect macrophage viability (Figure 4A,B), while the free-formed BAM15 (BAM15) at 75 ng/well was toxic to the cells (Figure 4C). Hence, BAM15 and BAM15 particles at a dose of 50 ng/well were used in subsequent experiments. Without LPS stimulation, blank particles, BAM15 particles, and BAM15 alone did not alter the production of M1 cytokines (TNF-α, IL-6, and IL-10) or the expression of M1 genes (*Il1b* and *Nos2*) (Figure 4D–H). Both BAM15 particles and BAM15 attenuated inflammatory responses of LPS-induced pro-inflammatory M1 macrophages as indicated by decreased levels of TNF-α, IL-6, and IL-10 and down-regulated *Il1b* and *Nos2* compared with the non-BAM15 groups (Figure 4D–H). For M2 markers, the blank particles, but not BAM15 particles and BAM15, and LPS downregulated *Arg1* expression (Figure 4I). Both BAM15 particles and BAM15 substantially increased *Arg1* expression upon LPS stimulation compared to the control LPS stimulation (Figure 4I). Moreover, BAM15 particles and BAM15 alone but not LPS enhanced *Tgfb* expression (Figure 4J). Upon LPS stimulation, BAM15 particles and BAM15 enhanced expression of *Tgfb* and *Fizz1* (Figure 4J,K). In the absence of LPS, BAM15 did not alter *Fizz1* expression, while blank particles and BAM15 particles downregulated *Fizz1* expression (Figure 4K). Furthermore, the impacts of BAM15 particles on mitochondrial energy metabolism were also investigated (Figure 4L–O). Consistent with the LPS-stimulated THP-1 macrophages (Figure 2B,E), the OCR and maximal respiration were decreased in RAW264.7 cells (Figure 4L,N). Although we could not observe alterations in glycolysis in LPS-stimulated THP-1 macrophages (Figure 2C,F), glycolysis was significantly elevated in LPS-stimulated RAW264.7 cells (Figure 4M,O). In the absence of LPS, blank particles, BAM15 particles, and BAM15 alone elevated mitochondrial energy (Figure 4L,N) and glycolysis (Figure 4M,O). Upon LPS stimulation, blank particles did not have an effect on mitochondrial energy and glycolysis, while BAM15 particles and BAM15 alone obviously enhanced mitochondrial energy and glycolysis (Figure 4L–O). Therefore, BAM15 particles and the free-formed BAM15 possibly restored macrophage function by altering mitochondrial energy metabolism.

**Figure 5:**
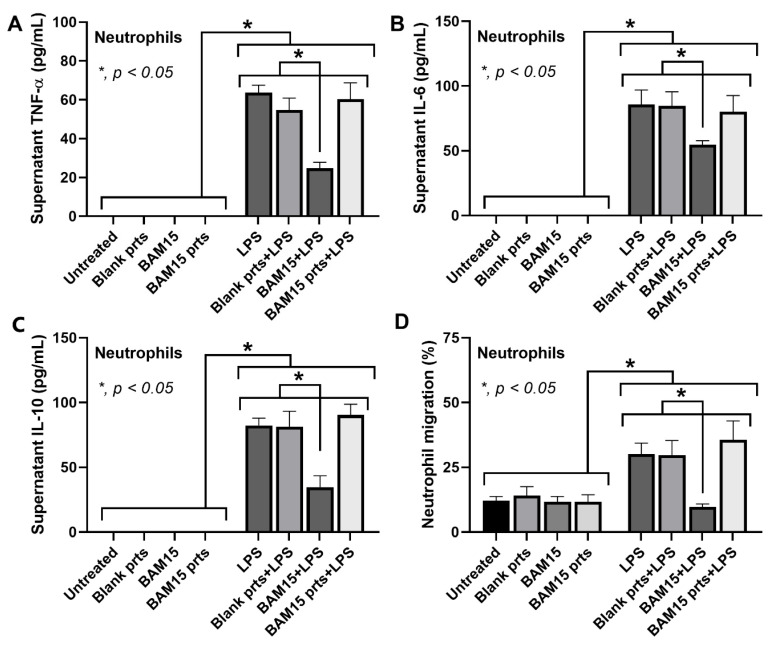
Characteristics of mouse peripheral blood neutrophils were determined 2 h after stimulation with culture medium (untreated), BAM15 (50 ng), BAM15 particles (BAM15 prts) (containing 50 ng of BAM15), and blank particles (with the same amounts as BAM15 particles) in the presence or absence of LPS. (**A**–**C**) The supernatant cytokines TNF-α, IL-6, and IL-10 were quantified by ELISA. (**D**) Neutrophil chemotaxis (cell migration) was demonstrated. The results were derived from three independent experiments. * *p* < 0.05 between the indicated groups.

Due to the important role of neutrophils at the inflammatory sites, neutrophil functions, including cytokine production and migration [32], were also tested (Figure 5). In the absence of LPS, blank particles, BAM15 particles, and BAM15 did not have any effect on neutrophils (Figure 5A–D). However, upon LPS stimulation, only the free-formed BAM15 but not blank particles or BAM15 particles suppressed the cytokine production and migration of neutrophils (Figure 5A–D). These results implied that the BAM15 particles may have a selectivity toward macrophages than neutrophils.

### 3.4. In Vivo Uptake of PLGA-FITC Particles by Macrophages

**Figure 6:**
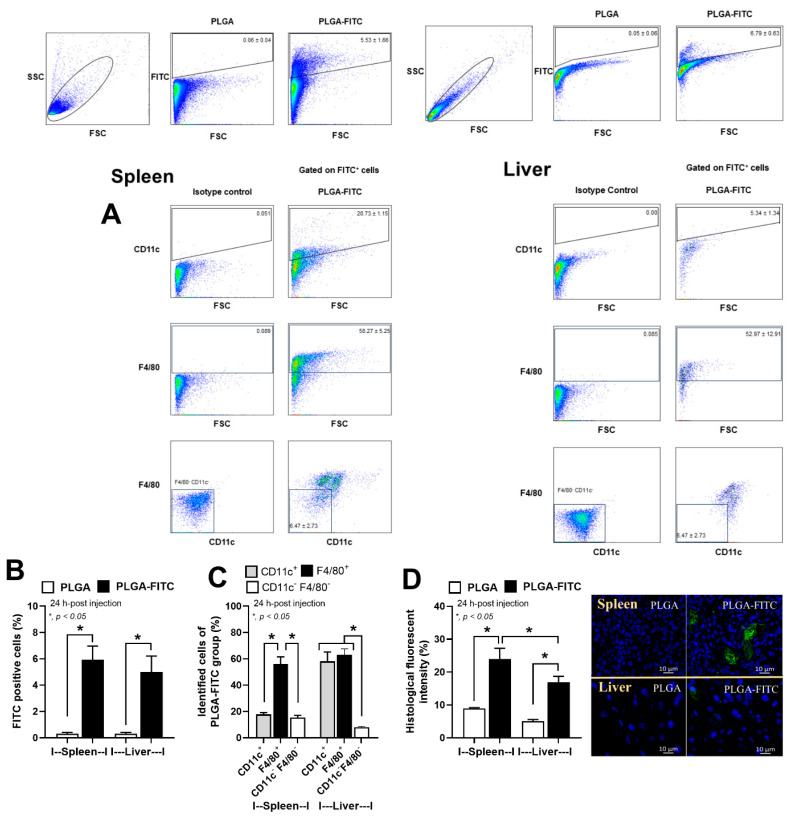
In vivo uptake of PLGA-FITC NPs. Unlabeled PLGA particles (PLGA), and FITC-tagged PLGA particles (PLGA-FITC) were administrated intraperitoneally to the intact mice. Twenty-four hours later, the spleens and the livers were collected, and splenic cells and liver cells were isolated for flow cytometric analysis. (**A**) Sequential identification of FITC^+^ cells, FITC^+^CD11c^+^ DCs, FITC^+^ F4/80^+^ macrophages, and FITC^+^ CD11c^−^ F4/80^−^ non-DC/macrophage cells in spleens and livers. (**B**) The proportion of FITC^+^ cells in the spleens and livers. (**C**) The proportion of DCs (CD11c^+^), macrophages (F4/80^+^), and non-DC/macrophages (CD11c^−^ F4/80^−^) in the FITC^+^ population. (**D**) The fluorescence imaging analysis of the FITC signal in spleens and livers. The bar graph indicates the fluorescence intensity of FITC. Representative images are demonstrated. The green color is the FITC signal, and the blue color is the DAPI staining. *n* = 4. * *p* < 0.05 between the indicated groups.

The uptake of particles by macrophages was investigated in vivo by intraperitoneal administration of FITC-tagged PLGA particles (PLGA-FITC particles) into healthy mice. The spleens and livers from mice with PLGA-FITC particles (tested group) or the blank particle (control group) were collected at 24 h after injection before the determination of FITC-positive (FITC^+^) cells using the flowcytometry markers for macrophage and dendritic cells (DCs), including F4/80 and CD11c, respectively (Figure 6A). The number of FITC^+^ cells apparently increased in both spleens and livers with the different gating results of CD11c^+^ cells in the spleens versus the livers (Figure 6A,B). As such, the splenic DCs express a wide range of CD11c as the CD11c^+^ signals are moderate to high (Figure 6A, left column) [35], while the conventional DCs in livers are a CD11c^high^ population (Figure 6A, right column) [36,37]. In both spleens and livers, PLGA-FITC particles were most abundant in F4/80^+^ cells (macrophages) but were less distributed in CD11c^+^ cells (DCs) and CD11c^−^ F4/80^−^ cells (non-DC/macrophage population), indicating that PLGA-FITC particles actively entered the macrophages (Figure 5C). The fluorescence signal of PLGA-FITC particles was also visualized by fluorescence imaging. The fluorescence signals of FITC were predominantly detected in the spleens and livers (Figure 5D), which confirmed our flow cytometric data (Figure 5B).

### 3.5. BAM15 Particles Attenuated Inflammation in LPS-Induced Sepsis Mice

**Figure 7:**
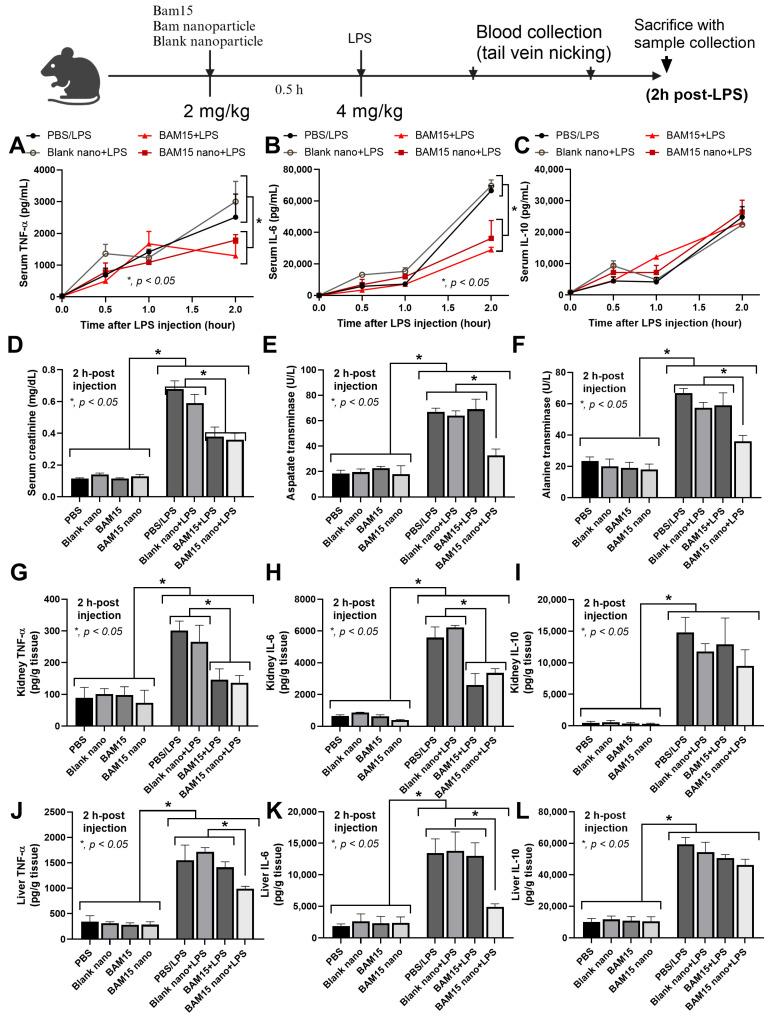
Schematic illustration demonstrating the LPS-induced sepsis mouse model. Mice received phosphate buffer solution (PBS), free BAM15 (BAM15) (2 mg/kg), BAM15 particles (BAM15 prts) (with BAM15 dose of 2 mg/kg), and blank particles (blank prts) (with the same amount of particles as BAM15 particles) 0.5 h prior to LPS injection. Blood was collected at 0.5 and 1 h after LPS injection. Mice were sacrificed 2 h after LPS injection, and blood and tissues (kidneys and livers) were collected. (**A**–**C**) The serum cytokines TNF-α, IL-6, and IL-10 were demonstrated at different time points. (**D**) The serum creatinine level was determined to analyze kidney function. (**E**,**F**) The levels of aspartate transaminase (AST) and alanine transaminase (ALT) were determined to analyze liver function. (**G**–**L**) Kidney and liver cytokines were also demonstrated. *n* = 5–7. * p < 0.05 between the indicated groups.

To prove the therapeutic potential of BAM15 particles in inflammatory disease, the mouse model of LPS-induced sepsis was used to test blank particles, BAM15 particles, and the free-formed BAM15 (Figure 7). There were neither inflammatory responses (serum cytokines) nor organ damage in the mice without LPS injection, including the PBS injection group, blank particles, BAM15 particles, and the free-formed BAM15 (Figure 7A–L). These data highlighted the non-obvious in vivo toxicity of blank particles, BAM15 particles, and BAM15 alone, despite the higher dose of BAM15 here compared with a previous publication [14]. With the sepsis model, the blank particles did not reduce inflammation and organ damage in LPS-injected mice (Figure 7A–L). In contrast, both BAM15 and BAM15 particles alleviated inflammation and organ damage in the LPS-injected mice as indicated by reduced serum inflammatory cytokines (TNF-α and IL-6 but not IL-10) and serum creatinine (Figure 7A–D). Additionally, liver enzymes, including aspartate transaminase (AST) and alanine transaminase (ALT), were highly elevated in LPS-injected mice, and these levels were attenuated by treatment with BAM15 particles but not the free-formed BAM15 (Figure 7E,F). The administration of BAM15 particles or BAM15 alone in LPS-induced sepsis mice attenuated inflammatory cytokines (TNF-α and IL-6 but not IL-10) in kidneys (Figure 7G,I). In sepsis mice, the production of liver TNF-α and IL-6 was also decreased by treatment with BAM15 particles but not BAM15, while the liver IL-10 level was not affected by all treatments (Figure 7J,L). Collectively, our data demonstrated that BAM15 particles had low toxicity in healthy mice, while the particles exhibited superior therapeutic efficacy in severe inflammatory disease when compared with the free-formed BAM15.

## 4. Discussion

Sepsis is a life-threatening severe inflammatory response that causes multiorgan failures and dysregulation of the host immune response, resulting in an increase in the mortality rate [38,39]. Pro-inflammatory M1 macrophages secrete a large amount of pro-inflammatory cytokines, which leads to the sepsis cytokine storm and subsequent hyperinflammatory septic shock [2]. Although the strategy of sepsis attenuation using macrophage targeting therapy is currently non-available, blocking the sepsis-induced cytokine storm through the inhibition of cytokine production from macrophages is an interesting hypothesis [38,39]. In this study, human monocytes (THP-1 cells) were differentiated with phorbol 12-myristate 13-acetate (PMA) into macrophages [30] before the induction of M1 polarization by LPS and M2 macrophages using IL-4 and cancer cell supernatants [31] (Figure 1A–H). However, only the supernatant from CaCO2 (colon) cells but not HepG2 (liver) cells induced M2-associated genes (Figure 1F–H), suggesting a different potency of various cancers in the activation of tumor-associated macrophages (TAMs; an M2-like polarization).

Oxidative phosphorylation (OXPHOS), an oxygen-dependent process, and glycolysis, an oxygen-independent process, are two major metabolic pathways that provide energy for cell function [40]. Due to the indispensable roles of mitochondrial metabolism in macrophage polarization [8], mitochondrial uncoupling agents play an important role in cell activities partly through cell energy regulation [11]. Here, the activators for both M1 (LPS) and M2 (cancer supernatants and IL-4) and BAM15 (a synthetic mitochondrial uncoupling agent) reduced mitochondrial energy (Figure 2A,B,E), perhaps to initiate specific responses. The activation by BAM15 and the HepG2 supernatant reduced mitochondrial function, despite the non-alteration in cytokines and expression of macrophage polarization genes, implying a positive activation, perhaps for other activities of the cells (Figure 2E). Indeed, macrophage cell energy, especially glycolysis, is important for responses against LPS (cytokine production) and cancers [41,42] partly because of the common hypoperfusion in clinical situations; for example, sepsis-induced vasodilatation and inadequate nourishment in the large tumors [3,43]. Despite the glycolysis preference of LPS-associated M1 macrophages and TAMs [44], the role of mitochondria in both M1 and M2 macrophages, at least in some situations, has been reported [32,45,46,47]. 

Here, in human macrophages (THP-1 cells), both activators of M1 (LPS) and M2 (cancer supernatant) macrophages reduced only mitochondrial activity without affecting glycolysis. Additionally, BAM15 interfered only with LPS-induced M1 polarization but not M2 polarization, as indicated by reduced cytokine levels and the decreased expression of M1-associated genes. In our study, BAM15 alone reduced maximal respiration (a mitochondrial activity) in macrophages (Figure 2E; untreated vs. BAM1), but the addition of BAM15 surprisingly improved the LPS-induced cell energy defect (Figure 2E; LPS vs. LPS + BAM15). This contradictory characteristic might be explained through the mitochondrial inhibitory property of BAM15 that starts with a short elevation in mitochondrial functions, followed by reduced mitochondrial activities [14]. The overwhelming responses to LPS (such as an overt cytokine production) without BAM15 might lead to mitochondrial destruction, while mitochondrial restoration with BAM15 might be able to preserve some mitochondria. Then, the preserved mitochondria might be able to restore the functions after the disappearance of the LPS effect. Notably, either BAM15 or LPS reduced mitochondrial activities but only LPS elevated macrophage supernatant cytokines, indicating the possible non-energy-dependent anti-inflammation of BAM15, for example, the upregulation of *Tgf-β*, an anti-inflammatory gene (Figure 4J) [48]. Hence, there are several anti-inflammatory mechanisms of BAM15 that may be useful for sepsis adjunctive therapy.

According to reports on the active capture of PLGA particles of 500 nm in size by macrophages [20] and the great biodistribution of PLGA particles in the tissues [49], the intraperitoneal injection of PLGA particles demonstrated the prominent capture in the spleens and livers (the reticuloendothelial system), perhaps by splenic macrophages (at the marginal zones [50]) and Kupffer cells (at the portal areas and hepatic sinuses [1,51]). The specific size and shape of BAM15 particles might be responsible for the more selective uptake by macrophages over neutrophils [33,52], which was possibly responsible for a less anti-inflammatory impact of BAM15 particles on neutrophils than macrophages. On the other hand, the more dominant impact of BAM15 particles against M1 than M2 macrophages might be due to the diverse impact of particle sizes [53], different molecular signals [54], and/or phagocytosis activities [55] between M1 versus M2. Although more mechanistic exploration of these topics is interesting, our project focused mainly on M1 inhibition in sepsis. 

Despite the previous use of BAM15 for sepsis attenuation [47], the high doses of BAM15 possibly cause hepatic damage, as previously reported [32,56]. Here, BAM15, at a high dose of free-form or BAM15 particles, was not toxic to the mice (Figure 7). Regarding the selective blockage only in macrophages, our particles might be an additional strategy to the conditional gene deletion (LyM-Cre) [24,32] and nanoparticle [25,26] strategies that have been previously described. With our previous protocol [20], PLGA particles were selectively delivered to macrophages but not DCs (Figure 6), and BAM15 particles had neutralizing effects on LPS-induced inflammation only in macrophages but not neutrophils (Figure 5). During a short period after LPS injection (within 2 h), the impact of BAM15 particles on macrophage responses was dominant. More importantly, the reduced toxicity of BAM15 particles compared with BAM15 alone was also demonstrated by the MTT assay (Figure 4A–C); however, both BAM15 and BAM15 particles showed a similar effect on M1 polarization in RAW264.7 macrophages (Figure 4) and in LPS-induced sepsis mice (Figure 7).

Regarding clinical translation, our results support the possibility of immune modulation in some conditions through the specific blockage of the limited types of immune cells. While general mitochondrial blockage in all cells with BAM15 can cause adverse effects in some specific cells (especially hepatocytes) [32,56], the targeted drug delivery to macrophages possibly helps to reduce off-target side effects. Although our study demonstrated the metabolic reprogramming in macrophages using the PLGA particle platform, several further adaptations to the particles are still needed to improve the selectivity and specificity of the particles. For future personalized medicine, biomarkers that demonstrate hyperfunction of major immune cells, including macrophages, neutrophils, or adaptive immune cells, in sepsis could be a target for particle delivery. Nevertheless, more studies are warranted. 

## Figures and Tables

**Table 1:** List of primers used in the study.

Name	Forward Primer (5′ to 3′)	Reverse Primer (5′ to 3′)
Arginase-1 (*Arg1*) (mouse)	CTTGGCTTGCTTCGGAACTC	GGAGAAGGCGTTTGCTTAGTT
Inducible nitric oxide synthase (*Nos2*) (mouse)	ACCCACATCTGGCAGAATGAG	AGCCATGACCTTTCGCATTAG
Interleukin-1β (*Il1b*) (mouse)	GAAATGCCACCTTTTGACAGTG	TGGATGCTCTCATCAGGACAG
Found in inflammatory zone 1 (*Fizz1*) (mouse)	GCCAGGTCCTGGAACCTTTC	GGAGCAGGGAGATGCAGATGA
Transforming growth factor-β (*Tgfb*) (mouse)	CAGAGCTGCGCTTGCAGAG	GTCAGCAGCCGGTTACCAAG
β-Actin (*Actb*) (mouse)	CCTGGCACCCAGCACAAT	GCCGATCCACACGGAGTACT
Arginase-1 (*ARG1*) (human)	CTTGGCTTGCTTCGGAACTC	GGAGAAGGCGTTTGCTTAGTTC
Inducible nitric oxide synthase (*NOS2*) (human)	ACCCACATCTGGCAGAATGAG	AGCCATGACCTTTCGCATTAG
Interleukin-1β (*IL-1β*) (human)	CCACAGACCTTCCAGGAGAATG	GTGCAGTTCAGTGATCGTACAGG
Found in inflammatory zone 1 (*FIZZ1*) (human)	GCAAGAAGCTCTCGTGTGCTAG	AACATCCCACGAACCACAGCCA
Transforming growth factor-β (*TGFB*) (human)	CAGAGCTGCGCTTGCAGAG	GTCAGCAGCCGGTTACCAAG
β-Actin (*ACTB*) (human)	CCTGGCACCCAGCACAAT	GCCGATCCACACGGAGTACT

**Table 2:** Hydrodynamic sizes and polydispersity index (PDI) and zeta potential values of particles.

Particles	Hydrodynamic Size (nm)	PDI	Zeta Potential (mV)
PLGA	527 ± 6	0.227 ± 0.009	−36.1 ± 0.3
PLGA-BAM15	559 ± 17	0.441 ± 0.147	−30.1 ± 0.5

## Data Availability

The data are contained within the article.

## Supplementary Materials

The following supporting information can be downloaded at: https://www.mdpi.com/article/10.3390/pharmaceutics15122695/s1, Figure S1: Absorbance of BAM15.
