# Supplementary material for: Polymeric Particle BAM15 Targeting Macrophages Attenuates the Severity of LPS-Induced Sepsis: A Proof of Concept for Specific Immune Cell-Targeted Therapy
Source: Pharmaceutics. 2023 Nov 28;15(12):2695. doi: 10.3390/pharmaceutics15122695 (PMC10747619; doi:10.3390/pharmaceutics15122695)
Supplement: Supplementary file 1 [file pharmaceutics-15-02695-s001.zip › pharmaceutics-2693183-supplementary.pdf]

Supplementary Information

Polymeric Nanoparticle BAM15 Targeting Macrophages Attenuates Severity of LPS-Induced Sepsis: A Proof of Concept for Specific Immune Cell-Targeted Therapy

Kanyarat Udompornpitak[1,2], Thansita Bhunyakarnjanarat[1,2], Wilasinee Saisorn[2,3], Chonnavee Manipuntee[4,5], Kittawat Plengplang[2], Samarch Sittichaitaweekul[2], Panisa Jenphatanapong[2], Suwasin Udomkarnjananun[6], Warerat Kaewduangduen[5], Kasirapat Ariya-anandech[5], Amanee Samaeng[5], Numpon Insin[4,5], Patcharee Ritprajak[5,7,#], Asada Leelahavanichkul[1,2,#]

[1]Department of Microbiology, Faculty of Medicine, Chulalongkorn University, Bangkok, Thailand

[2]Center of Excellence in Translational Research on Immunology and Immune-mediated Diseases (CETRII), Department of Microbiology, Faculty of Medicine, Bangkok, Thailand

[3]Interdisciplinary Program of Biomedical Sciences, Graduate School, Chulalongkorn University, Bangkok, Thailand

[4]Department of Chemistry, Faculty of Science, Chulalongkorn University, Bangkok, Thailand

[5]Research Unit in Integrative Immuno-Microbial Biochemistry and Bioresponsive Nanomaterials, Department of Microbiology, Faculty of Dentistry, Chulalongkorn University, Bangkok, Thailand.

[6]Division of Nephrology, Department of Medicine, Faculty of Medicine, Chulalongkorn University and King Chulalongkorn Memorial Hospital, Bangkok, Thailand.

[7]Department of Microbiology, Faculty of Dentistry, Chulalongkorn University, Bangkok, Thailand.

#Corresponding author

Asada Leelahavanichkul, MD., Ph.D., Associate Professor

Department of Microbiology, Faculty of Medicine, Chulalongkorn University, Bangkok, Thailand

E-mail: aleelahavanit@gmail.com

Tel: +66 2 256 4470, Fax: +66 2 252 5952,

Patcharee Ritprajak, DDS., Ph.D., Associate Professor

Department of Microbiology, Faculty of Dentistry, Chulalongkorn University, Pathumwan, Bangkok, 10330 Thailand

Email: Patcharee.R@chula.ac.th

Tel: +66-2-2188680

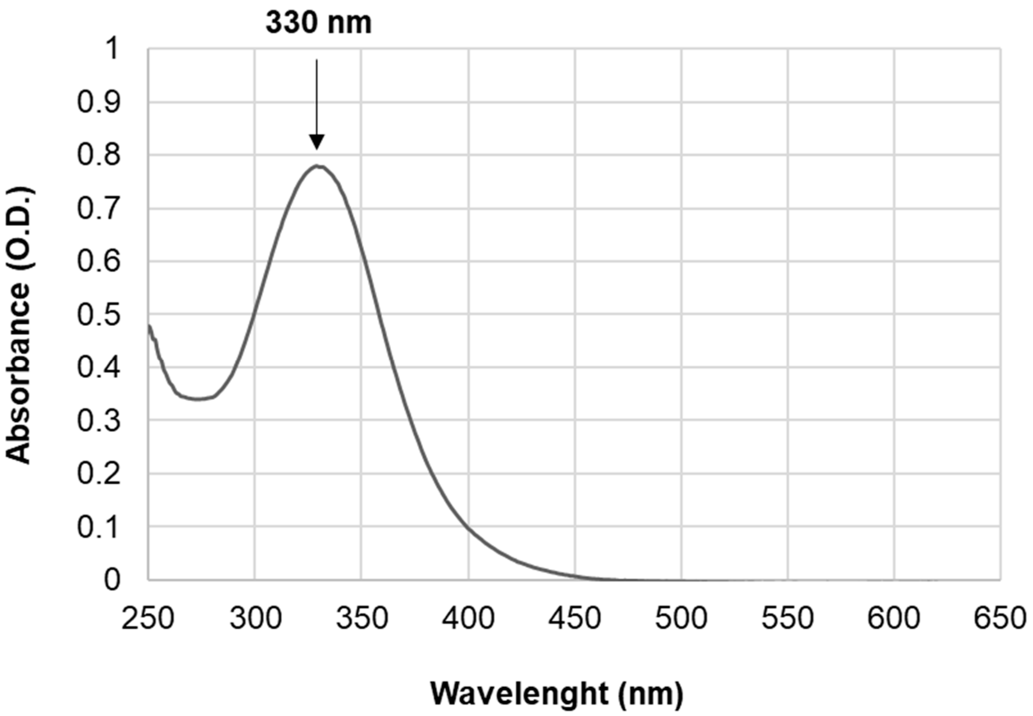

Supplementary Figure S1 Absorbance of BAM15

BAM15 solution was measure using UV-VIS spectrophotometry. The peak of absorbance was 330 nm.
